# Socioeconomic Status and Incident Type 2 Diabetes Mellitus: Data from the Women's Health Study

**DOI:** 10.1371/journal.pone.0027670

**Published:** 2011-12-14

**Authors:** Timothy C. Lee, Robert J. Glynn, Jessica M. Peña, Nina P. Paynter, David Conen, Paul M. Ridker, Aruna D. Pradhan, Julie E. Buring, Michelle A. Albert

**Affiliations:** 1 Division of Preventive Medicine, Department of Medicine, Brigham and Women's Hospital, Boston, Massachusetts, United States of America; 2 Division of Cardiovascular Medicine, Department of Medicine, Brigham and Women's Hospital, Harvard Medical School, Boston, Massachusetts, United States of America; 3 Department of Medicine, University Hospital, Basel, Switzerland; German Diabetes Center, Leibniz Center for Diabetes Research at Heinrich Heine University, Germany

## Abstract

**Objectives:**

We prospectively examined whether socioeconomic status (SES) predicts incident type II diabetes (diabetes), a cardiovascular risk equivalent and burgeoning public health epidemic among women.

**Methods:**

Participants include 23,992 women with Hb_A1c_ levels <6% and no CVD or diabetes at baseline followed from February 1993 to March 2007. SES was measured by education and income while diabetes was self-reported.

**Results:**

Over 12.3 years of follow-up, 1,262 women developed diabetes. In age and race adjusted models, the relative risk of diabetes decreased with increasing education (<2 years of nursing, 2 to <4 years of nursing, bachelor's degree, master's degree, and doctorate: 1.0, 0.7 [95% Confidence Interval (CI), 0.6–0.8], 0.6 (95% CI, 0.5–0.7), 0.5 (95% CI, 0.4–0.6), 0.4 (95% CI, 0.3–0.5); p_trend_<0.001). Adjustment for traditional and non-traditional cardiovascular risk factors attenuated this relationship (education: p_trend_ = 0.96). Similar associations were observed between income categories and diabetes.

**Conclusion:**

Advanced education and increasing income were both inversely associated with incident diabetes even in this relatively well-educated cohort. This relationship was largely explained by behavioral factors, particularly body mass index.

## Introduction

Type II diabetes (diabetes) is a potent risk factor for cardiovascular disease (CVD). Centers for Disease Control and Prevention (CDC) 2010 data show that almost 26 million Americans are diabetic, of whom 7.0 million remain undiagnosed [1]. Risk factors for diabetes include physical inactivity, family history of diabetes, impaired glucose metabolism and race/ethnicity. Although unhealthy lifestyle increases the risk of diabetes, and lower socioeconomic status (SES) is associated with higher CVD risk, whether diabetes, a CVD risk equivalent is similarly related to SES is uncertain [2].

To date, a majority of studies about SES and diabetes have been cross-sectional in nature. For example, low education level and occupational position were associated with a three-fold risk of prevalent diabetes in a cross-sectional cohort of middle aged men and women [3]. Similar findings were noted in the KORA Survey 2000 among elderly women [4]. In a prospective analysis utilizing the National Health and Nutrition Evaluation Study (NHANES) 1 Epidemiologic Follow up Study (NHEFS) data, higher income and education were associated with lower rates of diabetes in women [5]. Results from the prospective Whitehall II Study evaluating the association of social position with diabetes also indicate that among men and women lower civil service employment grade was related to at least a 2-fold increase in diabetes risk, while data from the geographically localized Alameda County Study also showed decreased diabetes risk with increasing years of education [6], [7].

Besides the relative paucity of prospective data about SES and diabetes, there remains a lack of comprehensive information about the biologic mediators of any potential relationship. Thus, although factors such as obesity, older age, family history of diabetes, hypertension, abnormal lipid and other CVD biomarker levels are linked to the development of diabetes, whether or not these same factors mediate any relationship between SES and incident diabetes is not known [8], [9]. Current public health strategies related to diabetes care support physical activity, diet, insulin and oral medications to control glucose levels. However, SES which although not traditionally thought of as modifiable risk factor for disease after a certain age is arguably a potentially modifiable via early implementation of targeted public health strategies for vulnerable populations such as provision of safe, clean space for physical activity as well as educational and job opportunities aimed at improving SES disparities. Because the effect of SES on diabetes development has not been adequately examined, we sought to 1) evaluate the association between education and income and incident diabetes; and 2) examine significant mediators of any potential relationship in a large United States cohort of female health professionals in an effort to inform public health strategies.

## Methods

### Study Population

Study subjects were participants in the Women's Health Study (WHS; N = 39,876), a randomized, placebo controlled trial that evaluated the effect of low-dose aspirin and vitamin E in the primary prevention of CVD and cancer. Study design details have been previously described [10]. Randomization commenced in February 1993 and participants were followed until March 2007. Socio-demographic baseline variables including education and income were collected using mailed questionnaires. Follow-up questionnaires to assess a variety of health outcomes, including diabetes, were sent every 6 months during the first year and yearly thereafter. Excluded from this analysis were 1,170 women with physician diagnosed or self-reported diabetes at baseline, 7 participants with pre-randomization CVD, 11, 856 women with missing laboratory data of interest, 280 women with baseline Hb_A1c_≥6% and 2,571 women with missing demographic data. Thus, 23,992 participants form the basis of this analysis.

Covariates of interest collected at baseline include age, self-reported race/ethnicity, baseline hypertension, body mass index (BMI), family history of diabetes, strenuous aerobic exercise, smoking, hormone replacement therapy (HRT), and alcohol consumption. Family history of diabetes reflects self-report of diabetes mellitus in a first-degree relative. Race/ethnic background is self-reported; the majority of women are white (94.8%). We did not perform race/ethnicity specific analyses for this prospective evaluation due to relatively smaller sample sizes for non-white groups.

### Laboratory Analyses

Total cholesterol (TC), high-density lipoprotein cholesterol (HDL-C), triglycerides, and direct low-density lipoprotein cholesterol levels (LDL-C) were measured in a Center for Disease Control and Prevention standardized laboratory. High-sensitive C-reactive protein (hsCRP) levels were measured using a validated assay (Denka Seiken, Niigata, Japan). Soluble-Intercellular Adhesion Molecule-1 (sICAM-1) was measured with an ELISA assay (R&D Systems, Minneapolis, Minn) that uses a quantitative sandwich enzyme immunoassay technique. Fibrinogen concentration was quantified with a Roche Diagnostics immunoturbidimetric assay on a Hitachi 917 analyzer using reagents and calibrators from Kamiya Biomedical Company (Seattle, Washington). Hemoglobin A1c levels were determined on an analyzer (Hitachi 911) based on turbidimetric immuno-inhibition using packed red blood cells (Roche Diagnostics). All blood samples were evaluated in a blinded manner and in duplicate.

### Socioeconomic Status

Participants were grouped into 5 categories of professional education beyond high school: <2 years of health professional education (HPE), 2–<4 years of HPE, a bachelor's degree(BS), a master's degree(MS), and a doctoral degree (doctor of philosophy and/or medical degree). Annual household income is reported in 6 categories of US dollars (≤$19,999, $20,000 to $29,999, $30,000 to $39,999, $40,000 to $49,999, $50,000 to $99,999, and ≥$100,000). These categories were chosen because annual household income (income) was reported in ranges of income, therefore participant income range was converted to the midpoint income for the respective reported range. Moreover, these categories are consistent with and standardized according categories previously utilized in published data regarding education and income in this WHS cohort [11].

### Incident Diabetes

Methods of ascertainment of diabetes in the WHS have been previously reported [12]. Briefly, all participants were asked annually “In the past year, were you newly diagnosed with diabetes mellitus?” Additionally, subjects provided the month and year of diagnosis. Confirmation of diabetes was conducted in a blinded fashion using the diagnostic criteria recommended by the American Diabetes Association [13]. All self-reported cases of diabetes were investigated by either telephone interview conducted by a physician or a previously validated self-administered questionnaire that inquired about symptoms, diagnostic testing, and use of diabetic medications [14], [15]. Only confirmed cases of diabetes as validated by methods described above were included in this analysis. Interviews and supplemental questionnaires compared to medical record review resulted in positive predictive values >90% [14]. Furthermore, the positive predictive value of the supplemental questionnaire was 99% (95%CI 97–100%) [14], [15].

### Statistical Analysis

Baseline clinical and demographic characteristics were categorized according to education level. Lipid, inflammatory marker, and Hb_A1c_ levels are reported as medians with their associated interquartile ranges. Because of skewness in the distribution of hsCRP, sICAM-1, and fibrinogen, these cardiovascular markers were log-transformed for regression analyses. Cox proportional-hazards models were constructed to estimate hazard ratios (HR) and associated 95% confidence intervals for incident diabetes. The effects of education and income on diabetes risk were examined separately as well as simultaneously. Several models were constructed: Model 1) age and race/ethnicity adjusted; Model 2) model 1+family history of diabetes; Model 3) model 2+hsCRP, sICAM-1, fibrinogen, HDL-C, LDL-C, total cholesterol (TC), triglycerides, BMI, exercise, alcohol, smoking, and HRT use, hypertension, and HbA1c (considered fully adjusted model). Referent groups were women with <2 years of HPE and/or income level (≤$19,999).

In order to examine potential mediators of any relationship between SES and diabetes, we created three a priori risk factor groups based on clinical knowledge, including behavior, lipid, and inflammation categories. The behavioral mediator category consisted of BMI, exercise frequency, smoking history, hormone replacement therapy (HRT) use, and alcohol intake. Since BMI is associated with physical activity, it was included in the behavioral mediator category. The lipid mediator category included TC, LDL-C, HDL-C, and triglycerides. The inflammatory biomarker category included hsCRP, sICAM-1, and fibrinogen.

We evaluated the contribution of each individual risk factor as well as the three risk factor groups to the observed association between SES and diabetes using the formula: (HR_base model_−HR_adjusted model_)/(HR_base model_−1)×100% [16]–[18].

**Figure 1:**
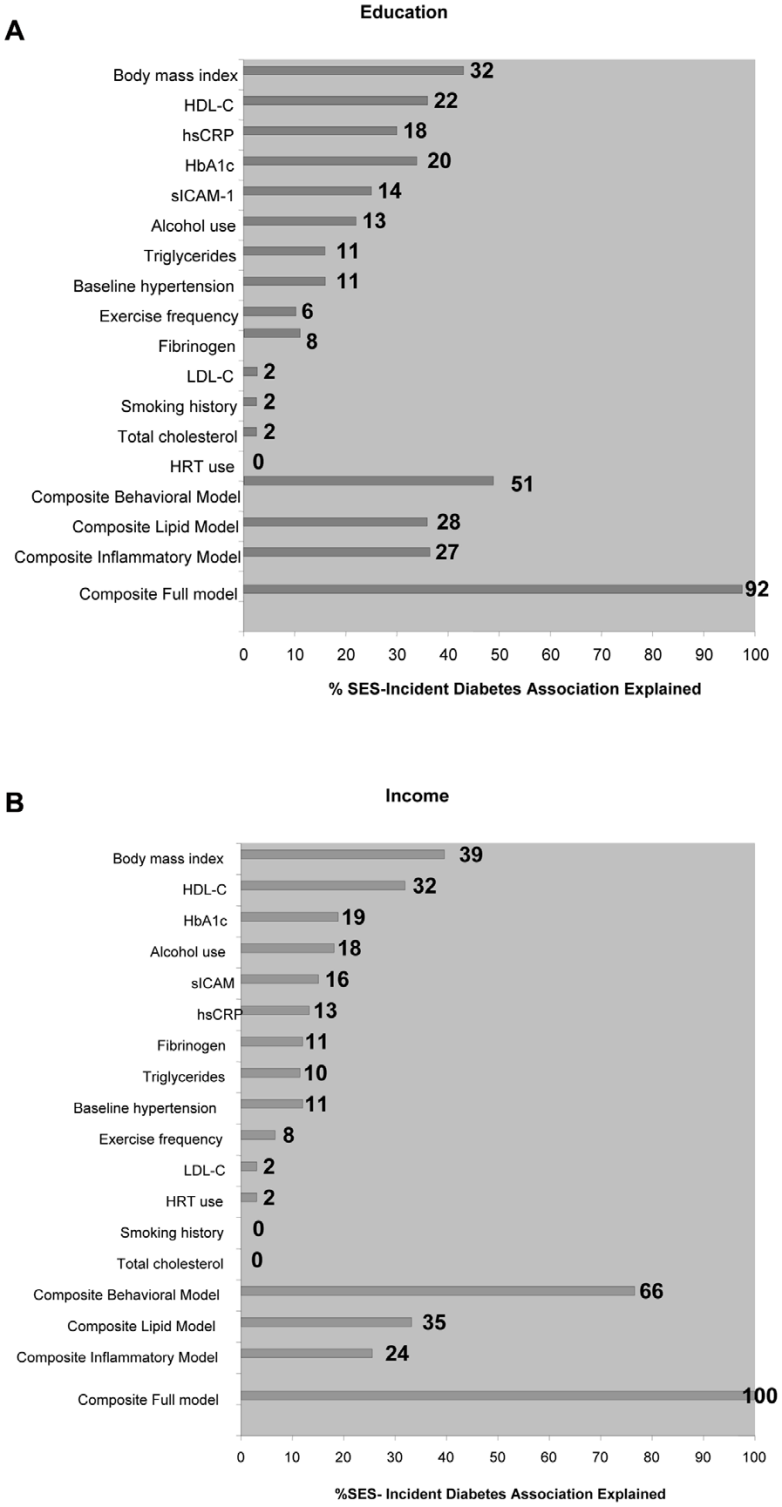
Percentage of socioeconomic status-incident diabetes association that is explained by mediators. The proportion of the risk attributable to increasing education levels; 1A) and for increasing income levels; 1B) that is explained by each mediator or set of mediators calculated as follows: (HR base model−HR adjusted model)/(HR base model−1)×100% [16]–[18]. HRs for the highest education or income categories compared with the HRs of the women with the lowest education or income without control for risk factors represented the base model whereas the adjusted model reflects control for each risk factor/risk factor category. Base model is adjusted for age, race/ethnicity and family history of diabetes.

Specifically, we evaluated the magnitude of change in the HRs for the highest education or income categories compared with the HRs of the women with the lowest education or income without (base model = adjusted for age, race/ethnicity and family history of diabetes) and adjusted for each risk factor/risk factor category (adjusted model) [15]. If the estimated hazard ratio in the base model was less than one while that in the adjusted model was greater than one, then we rounded the percent of the association explained by the mediators down to 100% (Figure 1).

Given the clinical association between diabetes and both BMI and physical activity, we created interaction terms to assess whether the effect of SES in predicting DM differed by BMI and exercise category [19], [20]. We assessed the significance of the interaction terms by comparing the likelihood ratio Chi-squared statistic with and without the interaction terms in the multivariable model. Tests for trend were performed using integer scores across categories. The proportional hazards assumption was examined by including a logarithm of time by education and income categories interaction [21]. We did not detect a violation of this assumption. All analyses were carried out using SAS version 9 (SAS Institute Inc., Cary, NC, USA). Two-tailed p<0.05 were considered statistically significant.

## Results

During a median follow-up period of 12.3±1.9 years, 1,262 participants developed diabetes (overall incidence was 4.5/1000 person-years). As shown in Table 1, compared to women with <2 years of HPE, women with doctorates were less likely to be obese (BMI, 24.3±4.2 versus 27.0±5.3 kg/m2 – not in table), have a history of hypertension and be current smokers. The most educated women were more likely exercise at least 4 times weekly and consume daily alcohol than the least educated women.

**Table 1:** Baseline Characteristics by Education Level.

	<2 HPE*	2–<4 HPE*	BS degree	MS degree	Doctorate
N = 23992	N = 2875	N = 10289	N = 5753	N = 3739	N = 1336
**Age (years)**	54.5±7.1	55.3±7.4	53.6±6.4	53.7±6.5	54.9±7.7
**Baseline Body Mass Index, kg/m2; %**					
≤25	42.0	51.3	55.2	57.6	65.1
25–29.9	34.9	31.3	30.3	28.4	25.1
≥30	23.1	17.4	14.6	14.0	9.8
**Exercise frequency, %**					
Rare/never	47.6	39.8	31.7	29.6	30.1
<1 time a week	18.9	19.6	20.7	19.0	17.8
1–3 times a week	26.5	30.4	35.2	36.6	35.0
4 times a week	7.1	10.1	12.4	14.8	17.1
**Hormone Replacement User (baseline), %**	41.0	44.9	44.1	43.4	44.6
**Family history of Diabetes, %**	28.2	25.1	23.1	22.7	24.5
**Baseline Hypertension, mmHg**					
(≥140/90), %	30.0	25.5	20.9	20.0	18.4
**Smoking history, %**					
Never	49.6	48.7	53.7	54.8	61.8
Past	30.8	38.1	37.5	38.6	32.6
Current	19.7	13.3	8.8	6.7	5.6
**Alcohol use, %**					
Rare/never	60.5	45.4	38.2	34.6	32.0
1–3 drinks per month	12.4	12.9	13.7	14.5	11.9
1–6 drinks per month	21.0	31.6	36.7	38.7	38.7
1 or more drinks per day	6.0	10.1	11.4	12.3	17.4
**Annual household income** **US $**					
≤19,999	16.1	4.6	2.4	1.5	0.9
20,000–29,999	25.0	10.4	5.4	3.9	1.8
30,000–39,999	18.7	16.3	12.1	8.61	4.4
40,000–49,999	16.8	18.3	16.5	14.8	6.7
50,000–99,999	22.0	41.5	48.5	52.1	32.9
≥100,000	1.3	8.9	15.1	19.2	53.2

*HPE indicates health professional education in years; all p_trend_ values<0.05 for demographic characteristics listed.

Table 2 shows the distribution of baseline lipid, inflammatory biomarker and hemoglobin A1c levels according to education and income levels. TC, LDL-C, and triglyceride levels decreased with higher education and income levels (p-trend<0.001 for each), whereas HDL-C levels increased with education and income categories (p-trend<0.001 for each). HsCRP, s-ICAM-1, and fibrinogen levels were all lower with higher categories of education and income (p-trend<0.001 for each).

**Table 2:** Median level of Lipids, Inflammatory and Markers Based on Education and Income Categories*.

Category	N	Total cholesterol (IQR), mg/dl	LDL(IQR), mg/dl	HDL(IQR), mg/dl	Triglycerides(IQR), mg/dl	hsCRP(IQR), mg/l	sICAM-1(IQR), ng/ml	Fibrinogen(IQR), mg/dl
**Education**								
**<2 HPE**	2875	211(186,240)	125(104,150)	49(41,59)	130(90,192)	2.5(1.0,5.1)	359(312,421)	364(317,417)
**2–<4 HPE**	10289	210(185,237)	122(102,145)	52(43,62)	123(86,178)	2.1(0.9,4.5)	346(304,397)	352(309,403)
**BS degree**	5753	205(181,233)	119(99,142)	53(45,64)	113(81,163)	1.8(0.7,3.9)	334(294,381)	344(304,393)
**MS degree**	3739	204(181,232)	119(98,141)	54(45,64)	108(76,157)	1.8(0.7,3.6)	332(292,377)	345(302,394)
**PhD/MD**	1336	206(183,230)	118(100,139)	56(46,65)	104(73,150)	1.4(0.6,3.3)	330(292,373)	340(301,388)
**Annual household income, US $**								
**<19,999**	1140	220(194,246)	132(110,156)	49(41,59)	134(98,195)	2.6(1.1,5.0)	371(325,430)	382(334,436)
**20,000–29,999**	2277	214(189,242)	127(106,151)	50(42,60)	132(92,194)	2.4(1.0,4.9)	357(314,411)	367(322,419)
**30,000–39,999**	3291	210(186,238)	124(102,148)	51(42,61)	125(88,178)	2.1(0.9,4.5)	351(307,403)	358(313,410)
**40,000–49,999**	3960	209(184,234)	122(101,144)	52(43,62)	120(85,173)	2.1(0.9,4.4)	344(302,396)	355(311,403)
**50,000–99,999**	10077	205(181,232)	119(99,141)	52(44,63)	113(81,168)	1.9(0.8,4.1)	336(295,384)	343(303,392)
**>100,000**	3247	203(181,230)	116(97, 138)	56(47,67)	103(74,152)	1.5(0.6,3.3)	325(287,367)	331(292,379)

*All values and levels are presented as median and the associated interquartile range (IQR).

For each parameter, P<0.001 (Kruskal-Wallis test).

Table 3 depicts the hazard ratios for diabetes according to education and income categories. We observed an inverse relationship between increasing education and incident diabetes. Adjustment for age, race/ethnicity, and family history (Model 2) was associated with relative risk reductions of 28%, 38%, 46%, and 63% with 2–<4 years of HPE, BS degree, MS degree, and doctorate respectively (p-trend<0.001). In a similar multivariable model (not shown in the Table) containing both education and income as independent variables, we observed a comparable trend; namely 21%, 30%, 38%, and 55%, relative risk reductions associated with 2–<4 years HPE, BS degree, MS degree, and doctorate degrees respectively (p-trend<0.001).

**Table 3:** Hazard ratios and Associated 95% CI for Incident Diabetes based on Education and Income.

Education	<2 HPE	2–<4 HPE	BS degree	MS degree	Doctorate		
N	2875	10289	5753	3739	1336		
Events	226	567	274	154	41		
Person-yrs	33240	121110	68287	44039	15431		
	Referent	HR(95%CI)	HR(95%CI)	HR(95%CI)	HR(95%CI)	**P trend**	
* Model 1	1.00	0.70(0.60,0.81)	0.59(0.50,0.70)	0.52(0.42,0.63)	0.36(0.26,0.50)	<0.001	
† Model 2	1.00	0.72(0.61,0.84)	0.62(0.52,0.74)	0.54(0.44,0.67)	0.37(0.27,0.52)	<0.001	
§ Model 3(Full Model)	1.00	0.96(0.83,1.13)	0.97(0.81,1.16)	1.02(0.83,1.26)	0.95(0.68,1.33)	0.96	

Annual household income, US $	≤$19,999	$20,000–29,999	$30,000–39,999	$40,000–49,999	$50,000–99,999	≥$100,000	
N	1140	2277	3291	3960	10077	3247	
Events	68	141	209	236	527	81	
Person-yrs	12836	26129	38454	46746	119291	38651	
	Referent	HR(95%CI)	HR(95%CI)	HR(95%CI)	HR(95%CI)	HR(95%CI)	**P trend**
* Model 1	1.00	1.01(0.76,1.35)	1.00(0.76,1.32)	0.92(0.70,1.21)	0.80(0.61,1.03)	0.37(0.26,0.51)	<0.001
† Model 2	1.00	1.01(0.75,1.35)	1.02(0.77,1.35)	0.94(0.71,1.24)	0.81(0.62,1.05)	0.38(0.27,0.53)	<0.001
§ Model 3(Full Model)	1.00	1.28(0.96,1.72)	1.41(1.07,1.86)	1.48(1.12,1.96)	1.40(1.07,1.83)	1.04(0.74,1.46)	0.42

*Age and race/ethnicity.

† **Age, race/ethnicity, and family history of diabetes.**

§ Age, race/ethnicity, family history of diabetes, hsCRP, sICAM-1, fibrinogen, HDL-C, LDL-C, total cholesterol, triglycerides, BMI, exercise frequency, alcohol consumption, smoking history, HRT use, hypertension, HbA1C.

In separate Cox regression models that considered each risk factor, one at a time, and adjusted for age, race/ethnicity, and family history of diabetes, there was minimal attenuation noted in the HR comparing the most educated to the least educated women based on education and income respectively (Appendix S1 A/B); body mass index contributed the largest effect of the individual factors. For education, when lipid, inflammatory, and behavioral mediators were considered simultaneously in a fully adjusted model, the HR comparing the most with the least educated women was substantially attenuated; increasing from HR 0.37 (95%CI 0.27–0.52) in the base model (same as model 2, Table 3) to HR 0.95 (95%CI 0.68–1.33) [p-trend 0.96]. In a similar evaluation, the HR comparing the highest with the lowest income level was substantially attenuated; increasing from HR 0.38 (95%CI 0.27–0.53) in the base model to HR 1.04 (95%CI 0.74–1.46), [p-trend 0.42]. As the inverse relation between SES and diabetes was essentially entirely explained by the mediators examined, we sought to evaluate the proportion of the relationship explained by each set of potential mediators (Figure 1).

Table 4 demonstrates hazard ratios associated with incident diabetes and education/income based on control for certain biomarkers of inflammation, lipids, behavioral characteristics and a fully adjusted model separately.

**Table 4:** Hazard ratios and Associated 95% CI for Incident Diabetes based on Education and Income.

Composite Models for Education	Referent<2 HPE	HR(95%CI)2–<4HPE	HR(95%CI)BS degree	HR(95%CI)MS degree	HR(95%CI)Doctorate	P trend	
†Inflammatory model	1.00	0.82(0.70,0.96)	0.78(0.65,0.93)	0.73(0.60,0.90)	0.54(0.39,0.76)	<0.001	
‡ Lipid model	1.00	0.85(0.73,0.99)	0.79(0.66,0.94)	0.75(0.61, 0.93)	0.55(0.40, 0.78)	0.001	
§Behavioral model	1.00	0.88(0.75,1.03)	0.88(0.74, 1.06)	0.82(0.66, 1.01)	0.69(0.49,0.96)	0.02	
¶Full model	1.00	0.96(0.83,1.13)	0.97(0.81,1.16)	1.02(0.83,1.26)	0.95(0.68,1.33)	0.96	

Composite Models for Annual household income, US $	Referent<$19,999	HR(95%CI)$20,000–29,999	HR(95%CI)$30,000–39,999	HR(95%CI)$40,000–49,999	HR(95%CI)$50,000–99,999	HR(95%CI)>$100,000	P trend
†Inflammatory model	1.00	1.05(0.78,1.40)	1.10(0.83,1.45)	1.05(0.80,1.39)	0.97(0.74,1.26)	0.53(0.38,0.74)	0.0003
‡ Lipid model	1.00	1.08(0.81,1.44)	1.18(0.89,1.55)	1.16(0.88,1.53)	1.06(0.81,1.38)	0.60(0.43,0.84)	0.009
§ Behavioral model	1.00	1.11(0.82, 1.48)	1.24(0.94,1.64)	1.18(0.90,1.56)	1.18(0.90,1.54)	0.79(0.56,1.10)	0.50
¶ Full model	1.00	1.28(0.96,1.72)	1.41(1.07,1.86)	1.48(1.12,1.96)	1.40(1.07,1.83)	1.04(0.74,1.46)	0.42

† Age, race/ethnicity, family history of diabetes, hsCRP, sICAM-1, and fibrinogen.

‡ Age, race/ethnicity, family history of diabetes, HDL-C, LDL-C, total cholesterol, and triglycerides.

§ Age, race/ethnicity, family history of diabetes, BMI, exercise frequency, alcohol consumption, smoking history, and HRT use.

¶ Age, race/ethnicity, family history of diabetes, hsCRP, sICAM-1, fibrinogen, HDL-C, LDL-C, total cholesterol, triglycerides, BMI, exercise frequency, alcohol consumption, smoking history, HRT use, baseline hypertension, HbA1c.

Finally, we found no evidence of an interaction between the effect of SES with BMI or physical activity (education_BMI_ p-value>0.7; education_exercise_ p-value>0.9; income_BMI_ p-value>0.2; and income_exercise_ p-value>0.5).

## Discussion

In this prospective cohort of female health professionals, we observed a progressive decrease in diabetes with increasing levels of education and income. Our results indicate that lower SES is associated with increased diabetes risk in women, even in this relatively well-educated cohort of health professionals. Notably, one concern about many SES-health analyses has been their focus on the lower boundaries of SES thereby possibly missing the existence of “the gradient” at all levels of SES in middle aged and older women. We also sought to understand the mechanism by which SES is associated with a decreased hazard of diabetes. Our data suggests that a majority of this effect is mediated by measured lipid, inflammatory, and behavioral characteristics. In particular, behavioral factors significantly affected the relationship between education/income and diabetes risk. Thus, at a public health level, interventions targeting these behaviors could substantially impact the risk of diabetes. Our data represent one of the few comprehensive, prospective studies of SES and incident diabetes. Additionally, our results extend previous work by assessing a large group of apparently healthy women at baseline and by examining potential mediators of the relationship between SES and diabetes.

Previous work such as the cross-sectional analysis by Agardh and colleagues revealed that fathers' middle income position and lower educational levels were more associated with diabetes in women than in men [3]. Based on these findings, the authors posited that social position is more persistently influenced by family background in women than in men. Prospective work from the NHANES involving 11,069 subjects (∼62% women) showed a lower risk of diabetes among women with increasing education [5]. Notably in NHANES, even after control for behavioral factors such as body size, physical activity, diet, smoking, and alcohol use, the SES-incident diabetes relationship was not entirely explained. Consistent with our findings, Maty et al. found that study participants with <12 years education had 50% excess risk of incident diabetes compared with those with more education (HR = 1.5; 95%CI 1.11–2.04) [7]; income was also not associated with increased diabetes risk. In the Maty et al. study, measures of adiposity also significantly impacted the education-diabetes relationship. Unlike our data, neither of these aforementioned studies adjusted for lipid and inflammatory factors.

Our analysis reveals that the relationship between education and diabetes was most affected by behavioral factors. BMI explained the majority of the SES- DM association explaining 32% of the education and 39% of the income effects respectively. Indeed, lower educational and financial resources are in part associated with more risky health behaviors, lower levels of social support and more adverse physical and environmental exposures [22]. For example, inadequate dietary, housing, transportation options can lead to weight gain with resultant dyslipidemia as well as chronic psychological stress. Specifically, poor housing and transportation options might be associated with unsafe physical neighborhood environments that include higher levels of violence, lack of sidewalks and parks, factors that would decrease the likelihood of resident recreational physical activity. Experimental evidence suggests that at the biological level these experiences which likely occur throughout the lifespan contribute to the development of insulin resistance, excessive inflammation, dysregulation of the hypothalamic-pituitary axis and sympathetic system overdrive over time [23].

The importance of lifestyle factors such as BMI in diabetes risk was also demonstrated in data from the Nurses' Health Study [20]. Indeed, at the molecular level, higher BMI is associated with elevated plasma levels of free fatty acids (FFA) which promote peripheral (muscle) insulin resistance [24], clinically deleterious lipid levels, and increased inflammation. For example, our research team has previously shown that markers of inflammation such as hsCRP are significant predictors of incident diabetes [25]. Likewise, in other work, Rathmann et al. demonstrated that adjustment for BMI and waist circumference resulted in a null association between increased CRP and low SES (p = 0.23) [26]. Extending previous work, our analysis here also investigates the impact of additional inflammatory markers including sICAM-1 and fibrinogen on the SES-diabetes risk relationship. However, these measured inflammatory biomarkers only partially explain our SES- diabetes relationship, an observation that is likely a consequence of the ability of these acute phase response biomarkers to capture only certain aspects of the inflammatory cascade.

Strengths of the present study include its prospective design, sample size, and evaluation of traditional and non-traditional risk indicators. The availability of long-term follow-up with a large number of confirmed events also enhanced our analysis. However, limitations also warrant discussion. First, the study population consisted of predominantly white, middle-aged female health professionals. This cohort consists of a low number of women from other race/ethnic groups, particularly African Americans and Hispanics, groups that have a much higher prevalence of diabetes raising the speculation that our findings could be more pronounced in these groups. To date, the largest prospective study of African-American women related to incident Type 2 diabetes showed that among 46, 382 women participating in the Black Women's Health Study (BWHS), women with ≤12 years relative to ≥17 years of education had a self-reported diabetes incident rate ratio of 1.28 (95% CI 1.15–1.43); for household income <$15,000 compared to >$100,000, the incident rate ratio for diabetes was 1.57. Similar to our study, the most important mediator in the relationship between SES and diabetes was body mass index. Notably, although BWHS participants have a broader education range in WHS, a majority of participants are college educated [27]. Second, a single baseline plasma measurement of each biomarker was utilized and thus we were unable to evaluate the effects of changes in plasma levels of inflammatory markers over time. Residual confounding by obesity and other unmeasured factors such as psychological stress is also possible. Third, we used BMI rather than waist circumference as the measure of obesity, however BMI and waist circumference both have similar demonstrated ability to predict diabetes [28]. Moreover individual body weight may change over time. Fourth, since it is plausible that diabetes risk factors accumulate over the lifespan, the WHS assessed only certain adult measures of SES [3], [29]. Fifth, we utilized education and income as measures of SES. Other measures of SES such as neighborhood composition/environment or occupation need to be incorporated into other longitudinal studies. Despite the high correlation between education and income and prior suggestions that education is a more robust measure of SES compared to other SES indicators, different measures of SES likely reflect different aspects of social stratification. Moreover, in the current study, it is possible that characteristics of retired women who would have lower income but potentially greater wealth than younger women could confound any multivariate income-diabetes relationship.

In summary, we found an inverse association between incident diabetes and increasing education and income levels in a large cohort of initially healthy, female health professionals. Our data are consistent with the hypothesis that these relationships are almost entirely mediated by lipid, inflammatory, and particularly by behavioral factors. Our findings extend existing data that suggest socioeconomic disadvantage at low SES boundaries predicts health risk to higher SES boundaries, an area where research is lacking. Finally, our results support the need for public health programs specifically targeting the obesity epidemic as a crucial means to decrease the incidence of diabetes even among well educated and affluent populations.

## Supporting Information

Appendix S1 **A/B: Mediation Analysis of individual and composite risk factors and their association with the SES-Incident Diabetes Relationship.**
(DOC)
