# Supplementary material for: Socioeconomic Status and Incident Type 2 Diabetes Mellitus: Data from the Women's Health Study
Source: PLoS One. 2011 Dec 14;6(12):e27670. doi: 10.1371/journal.pone.0027670 (PMC3237410; doi:10.1371/journal.pone.0027670)
Supplement: Appendix S1 — A/B: Mediation Analysis of individual and composite risk factors and their association with the SES-Incident Diabetes Relationship. (DOC) [file pone.0027670.s001.doc]

| **Appendix: Table 1A. Hazard ratios and Associated 95% CI for Incident Diabetes based on Education** | | | | | |  |
| --- | --- | --- | --- | --- | --- | --- |
| Education  N=23992 | <2 HPE  N=2875 | 2-<4 HPE  N=10289 | BS degree  N=5753 | MS degree  N=3739 | Doctorate  N=1336 | **P trend** |
|  | | | | | | |
| **Model** | Referent | HR(95%CI) | HR(95%CI) | HR(95%CI) | HR(95%CI) |  |
| *** Base model** | **1.00** | **0.72**  **(0.61, 0.84)** | **0.62**  **(0.52, 0.74)** | **0.54**  **(0.44,0.67)** | **0.37**  **(0.27,0.52)** | **<0.001** |
| +Baseline hypertension | 1.00 | 0.76  (0.65,0.88) | 0.67  (0.56,0.80) | 0.61  (0.49,0.74) | 0.44  (0.31, 0.62) | <0.001 |
| + HbA1c | 1.00 | 0.80  (0.69,0.94) | 0.71  (0.60,0.85) | 0.66  (0.54,0.81) | 0.50  (0.36,0.70) | <0.001 |
| **Inflammatory Mediators** | | | | | | |
| + hsCRP | 1.00 | 0.77  (0.66,0.90) | 0.72  (0.60,0.86) | 0.66  (0.54,0.81) | 0.48  (0.35,0.68) | <0.001 |
| + sICAM-1 | 1.00 | 0.80  (0.68,0.93) | 0.72  (0.61,0.87) | 0.66  (0.54,0.81) | 0.46  (0.33,0.64) | <0.001 |
| + Fibrinogen | 1.00 | 0.76  (0.65,0.89) | 0.68  (0.57,0.81) | 0.59  (0.48,0.72) | 0.42  (0.30,0.59) | <0.001 |
| **Lipid Mediators** | | | | | | |
| + HDL-C | 1.00 | 0.83  (0.71,0.97) | 0.76  (0.64,0.91) | 0.71  (0.58, 0.88) | 0.51  (0.37,0.72) | <0.001 |
| +Triglycerides | 1.00 | 0.75  (0.65,0.88) | 0.67  (0.56,0.80) | 0.62  (0.51,0.76) | 0.44  (0.32,0.62) | <0.001 |
| + LDL-C | 1.00 | 0.72  (0.62,0.85) | 0.63  (0.53,0.75) | 0.56  (0.45,0.68) | 0.38  (0.27,0.53) | <0.001 |
| + Total cholesterol | 1.00 | 0.72  (0.62,0.84) | 0.63  (0.53,0.75) | 0.55  (0.45,0.68) | 0.38  (0.27,0.53) | <0.001 |
| **Behavioral Mediators** | | | | | | |
| + Body mass index | 1.00 | 0.82  (0.70, 0.96) | 0.80  (0.65, 0.93) | 0.71  (0.57,0.87) | 0.57  (0.41, 0.80) | <0.001 |
| + Alcohol use | 1.00 | 0.78  (0.67,0.91) | 0.71  (0.60,0.85) | 0.64  (0.52, 0.78) | 0.45  (0.32, 0.63) | <0.001 |
| + Exercise frequency | 1.00 | 0.75  (0.64,0.87) | 0.67  (0.56,0.80) | 0.60  (0.49,0.74) | 0.41  (0.29,0.58) | <0.001 |
| + Smoking history | 1.00 | 0.72  (0.62,0.84) | 0.63  (0.52,0.75) | 0.55  (0.45,0.68) | 0.38  (0.27,0.53) | <0.001 |
| + HRT use | 1.00 | 0.72  (0.62,0.84) | 0.62  (0.52,0.74) | 0.55  0.45,0.67) | 0.37  (0.27,0.52) | <0.001 |
| **Composite Models** | | | | | | |
| † Inflammatory model | 1.00 | 0.82  (0.70,0.96) | 0.78  (0.65,0.93) | 0.73  (0.60,0.90) | 0.54  (0.39,0.76) | <0.001 |
| ‡ Lipid Model | 1.00 | 0.85  (0.73,0.99) | 0.79  (0.66,0.94) | 0.75  (0.61, 0.93) | 0.55  (0.40,0.78) | 0.001 |
| § Behavioral model | 1.00 | 0.88  (0.75,1.03) | 0.88  (0.74,1.06) | 0.82  (0.66,1.01) | 0.69  (0.49,0.96) | 0.02 |
| ¶ Full model | 1.00 | 0.96  (0.83,1.13) | 0.97  (0.81,1.16) | 1.02  (0.83,1.26) | 0.95  (0.68,1.33) | 0.96 |
| * Age, race/ethnicity, and family history of diabetes (Base Model)  † Age, race/ethnicity, family history of diabetes, hsCRP, sICAM-1, and fibrinogen  ‡ Age, race/ethnicity, family history of diabetes, HDL-C, LDL-C, total cholesterol, and triglycerides  § Age, race/ethnicity, family history of diabetes, BMI, exercise frequency, alcohol consumption, smoking history,  and HRT use  ¶ Age, race/ethnicity, family history of diabetes, hsCRP, sICAM-1, fibrinogen, HDL-C, LDL-C, total cholesterol,  triglycerides, BMI, exercise frequency, alcohol consumption, smoking history, baseline hypertension, HgA1c and HRT use | | | | | | |

| **Appendix: Table 1B. Hazard ratios and Associated 95% CI for Incident Diabetes based on Income** | | | | | | | |
| --- | --- | --- | --- | --- | --- | --- | --- |
| Annual household income, US $  N=23992 | <$19,999  N=1140 | $20,000-29,999  N=2277 | $30,000-39,999  N=3291 | $40,000-49,999 N=3960 | $50,000-99,999 N=10077 | >$100,000  N=3247 | **P trend** |
|  | | | | | | | |
| **Model** | Referent | HR(95%CI) | HR(95%CI) | HR(95%CI) | HR(95%CI) | HR(95%CI) |  |
| *** Base model** | **1.00** | **1.01**  **(0.75,1.34)** | **1.02**  **(0.77,1.35)** | **0.94**  **(0.71,1.24)** | **0.81**  **(0.62,1.05)** | **0.38**  **(0.27,0.53)** | **<0.001** |
| + Baseline hypertension | 1.00 | 1.07  (0.80,1.43) | 1.10  (0.84,1.45) | 1.03  (0.78,1.35) | 0.90  (0.69,1.17) | 0.45  (0.33,0.63) | <0.001 |
| + HbA1c | 1.00 | 1.06  (0.80,1.42) | 1.11  (0.84,1.46) | 1.11  (0.85,1.47) | 0.96  (0.74, 1.25) | 0.50  (0.36,0.70) | <0.001 |
| **Inflammatory Mediators** | | | | | | | |
| + hsCRP | 1.00 | 1.01  (0.75,1.35) | 1.04  (0.79,1.37) | 0.98  (0.74,1.29) | 0.87  (0.67,1.13) | 0.46  (0.33,0.64) | <0.001 |
| + sICAM-1 | 1.00 | 1.06  (0.79,1.41) | 1.11  (0.84,1.46) | 1.05  (0.80, 1.39) | 0.96  (0.73,1.24) | 0.48  (0.35,0.67) | <0.001 |
| + Fibrinogen | 1.00 | 1.06  (0.80,1.42) | 1.09  (0.83,1.44) | 1.02  (0.77,1.35) | 0.91  (0.70,1.18) | 0.45  (0.32,0.63) | <0.001 |
| **Lipid Mediators** | | | | | | | |
| + HDL-C | 1.00 | 1.07  (0.80,1.43) | 1.15  (0.87,1.52) | 1.15  (0.87, 1.52) | 1.03  ( 0.79,1.33) | 0.58  (0.41,0.80) | 0.002 |
| +Triglycerides | 1.00 | 1.01  (0.75,1.35) | 1.04  (0.79,1.38) | 0.98  (0.74,1.29) | 0.87  0.67,1.13) | 0.44  (0.31,0.61) | <0.001 |
| + LDL-C | 1.00 | 1.01  (0.76,1.36) | 1.03  (0.78,1.37) | 0.95  (0.72,1.26) | 0.83  (0.64,1.08) | 0.39  (0.28,0.54) | <0.001 |
| + Total cholesterol | 1.00 | 1.01  (0.75,1.35) | 1.03  (0.78,1.35) | 0.94  (0.71,1.24) | 0.82  (0.63,1.06) | 0.38  0.27, 0.53) | <0.001 |
| **Behavioral Mediators** | | | | | | | |
| + Body mass index | 1.00 | 1.07  (0.80,1.43) | 1.15  (0.87,1.52) | 1.07  (0.81,1.41) | 1.030  (0.79,1.34) | 0.62  (0.45,0.87) | 0.01 |
| + Alcohol use | 1.00 | 1.05  (0.78,1.40) | 1.11  (0.84,1.47) | 1.04  (0.79,1.38) | 0.95  (0.73,1.23) | 0.49  (0.35,0.69) | <0.001 |
| + Exercise frequency | 1.00 | 1.05  (0.78,1.40) | 1.08  (0.82,1.42) | 1.00  (0.75,1.31) | 0.88  (0.68,1.14) | 0.43  (0.31,0.60) | <0.001 |
| + Smoking history | 1.00 | 1.01  (0.75,1.35) | 1.02  (0.77,1.35) | 0.94  (0.71,1.24) | 0.82  (0.63,1.06) | 0.38  (0.27,0.53) | <0.001 |
| + HRT use | 1.00 | 1.02  (0.76,1.36) | 1.04  (0.79,1.38) | 0.96  (0.73,1.27) | 0.83  (0.64,1.08) | 0.39  (0.28,0.54) | <0.001 |
| **Composite Models** | | | | | | | |
| † Inflammatory model | 1.00 | 1.05  (0.78,1.40) | 1.10  (0.83,1.45) | 1.05  (0.80,1.39) | 0.97  (0.74,1.26) | 0.53  (0.38,0.74) | 0.0003 |
| ‡ Lipid Model | 1.00 | 1.08  (0.81,1.44) | 1.18  (0.89,1.55) | 1.16  (0.88,1.53) | 1.06  (0.81,1.38) | 0.60  (0.43,0.84) | 0.009 |
| § Behavioral model | 1.00 | 1.11  (0.82, 1.48) | 1.24  (0.94,1.64) | 1.18  (0.90,1.56) | 1.18  (0.90,1.54) | 0.79  (0.56,1.10) | 0.50 |
| ¶ Full model | 1.00 | 1.28  (0.96,1.72) | 1.41  (1.07,1.86) | 1.48  (1.12,1.96) | 1.40  (1.07,1.83) | 1.04  (0.74,1.46) | 0.42 |
| * Age, race/ethnicity, and family history of diabetes (Base Model)  † Age, race/ethnicity, family history of diabetes, hsCRP, sICAM-1, and fibrinogen  ‡ Age, race/ethnicity, family history of diabetes, HDL-C, LDL-C, total cholesterol, and triglycerides  § Age, race/ethnicity, family history of diabetes, BMI, exercise frequency, alcohol consumption, smoking history,  and HRT use  ¶ Age, race/ethnicity, family history of diabetes, hsCRP, sICAM-1, fibrinogen, HDL-C, LDL-C, total cholesterol,  triglycerides, BMI, exercise frequency, alcohol consumption, smoking history, baseline hypertension, HgA1c and HRT use | | | | | | | |
